# Costs of Early Stone Toolmaking cannot Establish the Presence of Know-how Copying

**DOI:** 10.1007/s12110-025-09494-w

**Published:** 2025-07-02

**Authors:** Claudio Tennie, William D. Snyder, Ronald J. Planer

**Affiliations:** 1 Faculty of Science, Department of Geosciences, Working Group Early Prehistory and Quaternary Ecology, University of Tübingen, Tübingen, Germany; 2 DFG Center for Advanced Studies “Words, Bones, Genes, and Tools”, Eberhard Karls University of Tübingen, Tübingen, Germany; 3 Senckenberg Centre for Human Evolution and Palaeoenvironment (HEP), Working Group Paleoanthropology, University of Tübingen, Tübingen, Germany; 4 School of Liberal Arts, University of Wollongong, Wollongong, Australia

**Keywords:** Cost–benefit analysis, Social learning, Epistemology, Stone toolmaking, Oldowan, Acheulean

## Abstract

Compared to other apes, humans show a distinctive capacity for the cultural learning and transmission of know-how: we extract know-how from other individuals and artifacts in ways that regularly give rise to forms of know-how that no single individual could realistically invent on their own. Such a capacity is plausibly foundational to humans’ striking cultural prowess and hence all that goes with it (e.g., symbolic language, religion). In this article, we critically examine attempts to date the transformation of know-how copying in the hominin lineage through an estimation of the *costs* of stone toolmaking. More specifically, we take as our target the idea that the costs inherent in making *early* stone tools, that is, Oldowan and Early Acheulean tools, already likely reflect a meaingful upgrade in hominin know-how copying abilities. Our survey of potentially relevant costs of stone toolmaking is generous, covering: (i) the risks and dangers of toolmaking; (ii) the time, energy, and opportunity costs of toolmaking; and finally (iii) the material costs of toolmaking. Ultimately, we find that, based on current evidence pertaining to these costs, the case for inferring know-how copying abilities in Oldowan or even Early Acheulean stone toolmakers is weak. This skeptical conclusion, combined with independent evidence that the design of stone tools during this period likely remained within the range of what the relevant hominins could invent without know-how copying, points to a later date for the establishment of this crucial human skill.

## Introduction

The majority of the prehistoric archaeological record is composed of stone tools – more precisely, knapped stone tools. Our prehistoric relatives, around three million years ago, began to make and use, especially sharp, knapped stone tools* in lieu *of their (shrinking) teeth (Harmand et al., 2015; McPherron et al., 2010; Schick & Toth, 1994; Toth & Schick, 2018). At first, these behaviors were likely sporadic, but they gradually increased in frequency within and between populations (and species) and eventually (some) hominin species became obligate knappers and knapped-tool users (perhaps around 300,000 years ago; see Shea, 2017).

Between 3.3 and 0.3 million years ago, knapped stone tools played an expanding role in hominin lives (Shea, 2011, 2017; Toth, 1985). Serious uncertainties exist, however, on several fronts. We must remain vigilant of ‘streetlight effects’ (*à la* the ‘drunkard’s search’ metaphor). Just as the proverbial drunk looks for his lost car keys next to a streetlight and not the location where he most likely lost them, we too may be overlooking some crucial areas. Whereas stone tools have largely survived to the present for us to observe (in the ‘streetlight’), organic material has usually fared more poorly, meaning that much of the rest of hominin behavior remains largely or entirely in the dark (the invisible organic tool age; e.g., Reindl et al., 2016; Rolian & Carvalho, 2017). Apart from indirect evidence from a few surviving bones with cut marks and potential early bone tools (e.g., Backwell & d’Errico, 2005; McPherron et al., 2010), the evidence of living non-human great apes (hereafter, apes; Bandini et al., 2022; Panger et al., 2002; Wynn & McGrew, 1989; Wynn et al., 2011), and fossil hominin anatomy (e.g., Kunze et al., 2024; cf. Dumoncel et al., 2021; Labra et al., 2023), we are bound to study knapped stones, particularly regarding cognition and social behavior.

Why does this matter? Firstly, attempting to understand past hominin behavior and lifeways is central to Paleolithic archaeology (Binford, 1972, 1977; Dibble et al., 2017; Schick & Toth, 1994). Secondly, based on lithic evidence, archaeologists draw a range of specific inferences about hominin behaviors and lifeways, many of which are questionable and, when taken together, contradictory. Recently, strong inferences have been made for the necessity or evolution of specific mechanisms for cultural transmission to mitigate the costs associated with making and using early knapped stone tools. One obvious type of cost stems from the nature of the tools themselves, namely: knapped stone tools cut. This was generally the intended effect of such tools – they would not have been useful otherwise. But at the same time, stone tool edges can and do unintentionally hurt their toolmakers (see Gala et al., 2023), implying that early stone toolmaking carried risks (e.g., Hiscock, 2014). But injury risks are just one of several types of costs that authors have suggested are relevant to early stone toolmaking; others include time, energy and opportunity costs, as well as costs relating to raw material procurement. As we will show, however, problems arise when trying to situate these costs into models that explain the evolution of (habitual versus obligate) tool-related behavior (Shea, 2017) and hominin cognitive and cultural abilities, especially regarding cumulative culture and know-how copying. Any attempt at evaluating this relationship would benefit first from a clearer understanding of both cultural transmission and the costliness of knapping.

## Cultural Transmission of Knapping Abilities?

Careful differentiation is needed when discussing cultural transmission. ‘Cultural transmission’ can be a troublesome term, because it might mislead readers into thinking that all social learning types produce copies of all aspects of an activity (see Buskell & Tennie, 2025; cf. Liu & Stout, 2023). This is not necessarily true, as social learning is not a monolithic category. Over the years, social learning has been divided and re-divided in various ways (see, e.g., Heyes, 2012; Lewis & Laland, 2012; Tennie et al., 2009, 2020). The end-result is a quagmire in the literature consisting of confusing and misleading nomenclature, contradictory application of terms, and deep misunderstandings.

Tennie and colleagues have recently sought to simplify the gnarly social learning nomenclature. In their framework, types of social learning are distinguished by the types of the information being transmitted: know-*where*, know-*when*, know-*who*, know-*how*, etc. (Bandini & Tennie, 2018; Planer et al., 2025; Tennie, 2023; Tennie et al., 2009, 2020). Each type of information also has a corresponding negative variety, e.g., social learning of know-where-*not*-to-hide. A key advantage of this framework is that it allows us to formulate hypotheses about what might distinguish human social learning from that of other apes in simpler, clearer, intuitive terms. For example, Tennie and colleagues propose that, while other apes are prodigious social learners of the know-*w* classes of information, humans show a special propensity – quantitatively, if not qualitatively different from other apes – for socially learning and transmitting know-*how* information, i.e., information related to behavioral and/or artifact forms. This is especially true where the know-how in question consists of long chains (Tennie et al., 2020; see also Enquist et al., 2023; Lind & Jon-And, 2024) and/or hierarchically structured forms (Tennie et al., 2020). In all or most cases, apes merely *trigger* latent know-how *development* in conspecific observers (see Sperber, 2000). This can either occur similarly to how one person’s laughter can cause another person to laugh, or, often, it works in conjunction with the social learning of other types of information (especially know-what and know-where information) and individual learning of other aspects (e.g., via trial-and-error or associative learning; Tennie et al., 2020; sometimes assisted by biological predispositions; see Enquist et al., 2023). The overall result is that affected individuals develop similar know-how, i.e., the main way in which ape cultures emerge and stabilize (compare Acerbi et al., 2022). Unlike humans, other apes rarely, if ever, spontaneously socially learn *supraindividual know-how* – i.e., know-how that could not be realistically innovated by an individual in a single lifetime (see Tennie et al., 2009, 2020; Tennie, 2023). Such know-how cannot merely be triggered (Buskell & Tennie, 2025).

Cultural transmission related to early knapped stone tools is currently a hot topic in lithic archaeology, especially within the subfield of evolutionary archaeology (e.g., Pargeter et al., 2019; Shea, 2011; Whiten, 2015). It is commonly proposed that stone tool manufacture was a crucial driver of the evolution of humans’ social learning abilities, leading to the development of know-how copying (e.g., T. Morgan et al., 2015a, b; Pargeter et al., 2023; Stout et al., 2008, 2010, 2019; van Schaik et al., 2019). This idea holds intuitive appeal; as any of us who have tried our hand at knapping know, the manufacture of, say, a thin, symmetrical handaxe is difficult. Even with the aid of expert instruction, it can take modern humans a long time to master such skills (Pargeter et al., 2019, 2020; cf. Cerasoni et al., 2023). On the other hand, the production of other, earlier stone tool forms (Table 1) is much more straightforward (e.g., Marzke & Shackley, 1986; Muller et al., 2022; Stout, 2011; Stout et al., 2015; cf. Shipton, 2020), at least from a manual point of view. To make an Oldowan tool requires little more than banging two stones together (albeit, the right type of stones, with suitable angles of blow; Li et al., 2023; Moore & Perston, 2016; Toth, 1985). So, in assessing the evolutionary relationship between stone tool manufacture and human social learning, we must first clarify *which type of technology* we are focusing on, and the respective types of social learning that a given technology demanded (e.g., know-*what*-to-knap versus know-*how*-to-knap).

**Table 1:** Breakdown of the hominin technologies that are implicated in the arguments below

Lithic industries	General character	Earliest known occurrence
Lomekwian	Large cores struck directly against static anvils (Lewis & Harmand, 2016)	ca. 3.3 Ma (Harmand et al., 2015)
Oldowan	Expedient knapping for flake production, often signified by pebble cores (Isaac, 1984; Toth, 1985)	ca. 2.6 Ma (Braun et al., 2019; Semaw et al., 1997)
Early Acheulean	Bifaces (most famously, handaxes) made from cores or large flakes (Isaac, 1972; Shea, 2013)	ca. 1.75 Ma (Lepre et al., 2011)
Late Acheulean	Larger, more uniformly-shaped bifaces, showing bilateral symmetry and reduced thickness. Diversification of knapping behavior to include, e.g., platform preparation and more frequent use of bone and antler retouchers (Nowell & White, 2010; Stout et al., 2014)	ca. 0.7 Ma (Roche, 2005; Stout, 2011)

Many researchers either explicitly or implicitly implicate *early stone tools* (Table 1) in arguing for evolutionary advances towards human-like social learning abilities (e.g., beyond an ape model; see Snyder et al., 2022; Tennie et al., 2016, 2017; Toth & Schick, 2018; Wynn & McGrew, 1989). Consequently, we target Oldowan and Early Acheulean tools, whereas we exclude Lomekwian tools (Harmand et al., 2015), due to the meager sample size of such pre-Oldowan data, despite pre-existing attempts to interpret the cognitive implications of Lomekwian artifacts (e.g., Lewis & Harmand, 2016).

We can suspect that hominin knapping involved social learning (e.g., Stout & Semaw, 2006) because it is widespread among – especially – apes (see Bandini & Tennie, 2017; Buskell & Tennie, 2025; Tennie et al., 2020), but apes seemingly lack know-how copying abilities like humans have (see above). Thus, social learning abilities *sensu lato* cannot automatically be equated with an ability to copy know-how (see Bandini et al., 2020; Tennie et al., 2009, 2020). As a baseline, we shall assume that hominin learning repertoires included (at minimum) the same set of social learning abilities in active use today in apes (know-*w* social learning and know-how *triggering*; e.g., Snyder & Tennie, 2023; Snyder et al., 2022; Tennie, 2023; Tennie et al., 2016, 2017). The interesting question is when, why, and how this ape-like portfolio of social learning was upgraded with know-how copying. This remains controversial (cf. Lycett, 2019; Shipton, 2020, 2024; Snyder et al., 2022; Sterelny & Hiscock, 2024; Tennie et al., 2016, 2017; Whiten, 2015; Wynn et al., 2011).

Only as we move forwards in time do such claims become progressively less controversial; no one doubts, for example, that Late Pleistocene multi-component projectile weapons, like the bow and arrow, demanded refined social learning skills (see, e.g., Lombard, 2024). In fact, the social learning requirements for knapping likely changed over time, due to (interlinked) changes in the species-specific social learning abilities and changes in the demands imposed by different stone tool types. The social learning demands of a specific artefact are themselves *relative* to the species-specific abilities and their environmental and cultural setting (Tennie & Hedwig, 2009; Tennie et al., 2020). For example, an Early Acheulean handaxe was theoretically beyond the individual reach of *Sahelanthropus tchadensis* in the inhabited contexts in their time (i.e., via a combination of triggering, individual learning, and social learning of know-*w*), but may have been individually reachable for some *Homo erectus* populations (Tennie et al., 2016, 2017, 2020). The relationship between tool making and social learning is bilaterally complex - not to mention also the additional roles of cologies and niches - without even implicating the implicit knapping costs.

### The Origins of Know-how Copying

Two broad types of reasoning are applied in this area. One line of reasoning identifies *artifact traits* that are *best explained* by the involvement of know-how copying in order to roughly date or map out a timeline of the evolution of such social learning (i.e., trait-derived estimation of human-like know-how copying’s origins; hereafter, *trait-derived estimation*). The second invokes other factors, e.g., the *impact of knapping costs* on hominin fitness, to infer (some degree of) *selection pressure on* learning abilities, especially for the evolution of know-how copying (i.e., cost-driven estimation of human-like know-how copying’s origins; hereafter, *cost-driven estimation*). These lines of thought are not mutually exclusive; both contribute to our understanding of biological and cultural evolution in our lineage, and both contain numerous competing models. In this article, we will only touch upon the former, instead focusing our critical attention mainly on the latter.

Although trait-derived estimation is not our current target, we will still briefly summarize. Here, an artifact type, toolmaking technique, or sequence is identified and argued, e.g., to be ‘too complex’ or ‘too cognitively demanding’ for a hominin to have invented on their own in a single lifetime (e.g., Shipton, 2020). Likewise, an archaeological trend where a specific form is preserved over a longer time span, e.g., variable stasis (or stases) in the Acheulean, is argued to be impossible without know-how copying (cf. Lycett, 2008; Lycett & Gowlett, 2008; Shipton, 2010, 2020; Shipton et al., 2021). We have countered such arguments in detail elsewhere (see, e.g., Andersson & Tennie, 2023; Planer et al., 2025; Snyder & Tennie, 2023; Snyder et al., 2022; Tennie, 2023; Tennie et al., 2016, 2017). In our view, there is strong empirical and theoretical justification for thinking the manufacture of early stone tools did not *require* know-how copying (an overview of the know-how social learning types that have been implicated in stone toolmaking in Table 2), but instead fell within the relevant hominins’ ‘zone of latent solutions’ (ZLS), i.e., that such forms demanded only (latent) know-how that was, in principle, individually innovatable and the frequency of which influenced by know-*w* social learning (otherwise, ‘minimal culture’; Neadle et al., 2017) and/or know-how triggering (Tennie et al., 2020). These minimal cultures can be just as varied as the different types of know-*w* social learning and know-how triggering that contribute to them (Table 3), with even greater variation arising from all the possible combinations in mixed learning pathways (e.g., social learning of know-where plus social learning of know-what and individual learning of know-how; see sections below). This approach is more parsimonious, as we do not need to assume the presence of learning abilities that are fully or largely absent in living apes (see above).

**Table 2:** Know-how copying social learning types (in ascending order according to approximate cognitive ‘complexity’) that have been *implicated* as being necessary for producing early stone tools or under selection in relation to early stone tools (*at least* one technique, method, or artifact form). For clarity’s sake, know-how copying needs to be defined and separated from know-how triggering (for the latter, see Table 3). Combinations are possible

Types of know-how copying	Defined as…	References
Type of copying inferred or otherwise unspecified		e.g., Eren et al., 2020; Shipton, 2020; Stout & Chaminade, 2007; Stout & Semaw, 2006; Stout et al., 2010; Wynn et al., 2011
Copying via emulation	Copying of environmental results (Tennie et al., 2010). At least some types of emulation can transmit know-how copies (Caldwell & Millen, 2009; Reindl et al., 2017)	e.g., T. Morgan et al., 2015a, 2015b; Toth & Schick, 2018
Copying via imitation	Typically, copying of the specific form of a behavior(al trait), otherwise known as action copying (Bandini et al., 2020; Neadle et al., 2017; Tennie et al., 2020)	e.g., Caruana et al., 2013; Lombao et al., 2017; T. Morgan et al., 2015a, 2015b; Schick & Toth, 1994, 2018; Shipton, 2010; Shipton & Nielsen, 2015; Stout & Khreisheh, 2015
Teaching of know-*how* copies	Modification of a model’s behavior, *in order to* facilitate or allow the know-how copying of another individual	e.g., Lombao et al., 2017; Stout & Khreisheh, 2015
(Proto-)language (especially as a medium to transfer know-how copies; see Dean et al., 2012)	Gestural linguistic transmission of know-how copies	e.g., Cataldo et al., 2018; Gärdenfors & Högberg, 2017; Putt et al., 2014 (for Early Acheulean)
	Verbal linguistic transmission of know-how copies	e.g., Lombao et al., 2017; Lucas et al., 2020

**Table 3:** Some of the main social learning types that do not produce know-*how* copies on their own. For readability, not all existing terms are added

Type	Definition	Examples for hominins and non-hominins
Know-how triggering	Observation of know-how *X* triggers the release or development of similar know-how *X* in observers – who could in principle have arrived at know-how *X* on their own (thus, *intra*-individual know-how; see Fehér et al., 2009; Sperber, 2000; cf. Corbey et al., 2016) This can range from triggering of highly biologically ‘pre-loaded’ know-how to know-how that is *partially* biologically ‘pre-loaded’ and (more or less) *partially* individually learned	Examples from primates – ordered from ‘more’ to ‘less’ biologically ‘pre-loaded’ (alternatively, from ‘less’ to ‘more’ individually learned) – include scratching contagion in lemurs and monkeys (Feneran et al., 2013; Padilha Lemes & Amici, 2024; Valdivieso-Cortadella, et al., 2023); leaf swallowing in chimpanzees and bonobos (a self-medicative behavior; Menzel et al., 2013); nettle feeding and food cleaning in gorillas (Neadle et al., 2017; Tennie et al., 2008); and nut-cracking in orangutans (Bandini et al., 2021a) Although such behaviors would not be preserved in the archaeological record, examples for know-how triggering would likely be similar to the primate cases, perhaps even somewhat exceeding the depth of know-how required for these behaviors
Know-where social learning	Classically called local enhancement (Heyes, 1994) – observers become more attracted to location *X* due to observation of models acting at/near *X*. The relative need for know-where social learning can differ for different locations (Tennie et al., 2009)	This is widespread among animals, including in apes with regards to, e.g., food location cues (Itakura et al., 2001) For stone knapping hominins, socially learning where to find suitable stone raw materials or where best to knap (e.g., Caruana, 2020; Hayden, 2008; Snyder & Tennie, 2023; Toth, 1985)
Know-what social learning	Classically called stimulus enhancement (Heyes, 1994; Zuberbühler et al., 1996) – observers become more attracted to stimulus *X* due to observation of models’ interaction with *X*. Sometimes stimulus enhancement is needed to overcome an otherwise negative stimulus (e.g., as in eating nettles; see Tennie et al., 2020)	Widespread type of social learning in animals, including in apes (e.g., for arbitrary food preferences; Shorland, et al., 2019), macaques (e.g., for stick tool use: Zuberbühler et al., 1996) and capuchin monkeys (e.g., for selecting nut cracking stones: Fragaszy et al., 2013; Ottoni & Mannu, 2001) For stone knapping hominins, what stone types to use or what parts of a carcass to process (e.g., Snyder & Tennie, 2023)
Other know-*w* social learning types	Additional cases where subjects may derive at *w* (e.g. when, what-*not*, etc.) partially or fully due to models	Good examples of this in other animal species include the learned avoidance of certain foods (know-what-not to eat) in blackbirds (Mason & Reidinger, 1982). There is comparatively little research on this in primates, but for an example see McLendon and Amoroso (2020) Hominins may have learned socially also what-*not* to eat (e.g., by observing disgust reactions in others)
Teaching of know-*w*	Any case where the model is somehow actively facilitates transmission of know-*w* information and triggering of know-how (Hoppitt et al., 2008). This includes (simple) scaffolding (Stout, 2005; Thornton & Raihani, 2010) The variation in teaching types corresponds to the different types of know-*w* social learning (i.e., there can be teaching of and/or via know-what, know-where, etc.; Hoppitt et al., 2008)	Teaching is generally rare in contemporary primates (Moore & Tennie, 2015), with most reports being anecdotal. The rare cases of teaching in primates seem to involve know-w social learning, but not know-how copying Some possible examples include the cultural transmission of know-what (such as teaching in chimpanzees: Musgrave et al., 2016) and supposed slowing down of actions of a mother macaque to facilitate social learning of dental floss know-what in her offspring (Watanabe et al., 2007) Teaching of know-*w* can also relate to dangerous stimuli. For example, wild meerkats teach offspring to hunt not by teaching them the know-how, but by providing them first with safe variants of know-what-to-hunt: scorpions with their stingers removed (Thornton & McAuliffe, 2006)

Because trait-based estimation has already been discussed in considerable detail elsewhere, from here on, we will discuss the costs of stone toolmaking (thus, cost-driven estimation). For example, according to one popular discussion point, stone tool manufacture is *dangerous*, and hence we ought to expect the early presence of – and/or selection for – know-how copying and/or teaching of know-how (Table 2) for mitigating these risks (e.g., Gala et al., 2023; Hiscock, 2014; Kovach & Gill, 2024; Lycett et al., 2015, 2016). Even if artifact forms are simple enough for members of a given species to come about via, e.g., know-*w* social learning (Table 3; see Gala et al., 2023; Lycett, 2019), the inherent risks are ostensibly important enough that they need to be reduced by deploying know-how copying of some kind(s) and in some way(s) (e.g., via teaching *how*-not-to-knap).

But again, there are significant doubts that the presence, let alone necessity, for know-how copying with regard to early stone tools can be demonstrated based exclusively on knapping costs, not least because many different, equifinal processes (alone or in combination) could have produced the evidence we find today. We elaborate on these doubts in the sections that follow.

### Cost-Driven Estimation of Know-how Copying’s Origins

As a first step, we note that, despite their increasing prominence in the literature, the cost-mitigating accounts are seldom sharply formulated and even more seldomly tested against *all* available evidence and *all* thinkable possibilities. To more clearly evaluate how the costs of knapping could have affected the emergence and development of specific social learning abilities, it is necessary to formulate (the differences between) the main models concerning this relationship and to clearly outline the base assumptions that contribute to these models.

The first assumption is that hominins were capable of social learning of some type. As explained above, we take this to be true. Another base assumption is that, under certain conditions, know-how copying was theoretically more beneficial than other social learning types, including in mitigating (some of) the costs of knapping. This seems fairly reasonable. If, for instance, someone were to become a firefighter – a dangerous job – that person would almost certainly benefit from copying experienced firefighters, who in turn acquired their firefighting skills from previous links in the chain of cultural evolution. Likewise, being able to copy supraindividual know-how was clearly beneficial for Inuits learning how to build sea-worthy kayaks (e.g., mitigating the risks of sinking; Boyd & Richerson, 2007). That know-how copying abilities can have payoffs, including in mitigating costs, is, of course, a rather trivial point. A bigger concern is this: unless the hominins that produced early stone tools actually were capable of any know-how copying variant (Table 2), the benefits of know-how copying cannot possibly be used to prove that hominins learned to knap via know-how copying. A hominin can hardly reap the benefits of an ability that it is incapable of even employing. As such, assuming *a priori* that hominin knapping relied on know-how copying abilities can lead to circular reasoning about the relationship between the benefits of know-how copying and the costs of knapping.

Numerous models describe the relationship between knapping costs and the evolution of social learning, particularly know-how copying. To impose much-needed order, we distinguish between the main classes of cost-driven estimation models. From the strongest to weakest implication of know-how copying, we would divide them into the categories as follows:

- *1. Cost-driven necessity models* assume that the mitigation of knapping costs was paramount, and therefore, (one or more types of) know-how copying was necessary to mitigate said costs.
- *2. Cost-driven selection pressure models* assume that know-how copying would have aided in mitigating costs, would thus have been adaptive (i.e., providing a fitness advantage), and would have been selected for. This model remains, however, agnostic concerning the when and how of the appearance of know-how copying abilities. As such, we can subdivide this category further.
- *2a. Early cost-driven selection pressure models* assume that the selection gradient began earlier, i.e., that hominins already employed (some kind of) know-how copying abilities for the production of early stone tools and that subsequent evolutionary processes acted upon these pre-existing abilities for producing these and/or subsequent types of stone tools.
- *2b. Late cost-driven selection pressure models* do not assume that there was already know-how copying of stone tool making skills for the earliest stone tools, but that the knapping costs, among other factors, would have started a selection gradient that led to the de novo evolution of know-how copying skills and/or its employment in stone tool production for later technologies.

Before we compare these cost-driven estimation models, however, we must first *attempt* to develop a better understanding of the *costs*, including mapping out what these costs were, what their relative value (e.g., for fitness) was, and the ways in which the specific costs might have been mitigated. By doing so, we can evaluate the (proposed) logical linkage between knapping costs and specific types of social learning and estimate when (but *not* a specific point in time; see Pargeter et al., 2023) knapping costs might have driven the evolution of human cultural transmission abilities from more-or-less ape-like social learning portfolios towards the development of – and eventual dependence upon – frequent and deep-reaching know-how copying (cf. Andersson & Tennie, 2023; Davidson & Noble, 1993; Whiten, 2015; Wynn & McGrew, 1989; Wynn et al., 2011).

## The Costliness of Knapping

It is clear that knapping could have had a variety of costs that are implicated in the expression of the behavior(s), but despite their likely *relevance* for knapping by prehistoric hominins, it is difficult to measure these costs, because they are intrinsically linked to assumptions about behavior frequency, survival dependence, and hominin biological and/or cognitive abilities (see, e.g., Karakostis, 2023; Shea, 2017; Toth, 1985). On a related note, the literature often assumes high fitness or survival costs from, e.g., *not* knapping at all, *not* mastering the knapping skillset *quickly enough*, or being a ‘bad’ knapper. This leads to potentially circular arguments for the necessity for know-how copying or steep positive selection for expedient learning of knapping skills (here also presupposing improved expediency with know-how copying). Similarly, it is hard to relate these costs to other factors, including, foremost, the *benefits* of knapping (which we do not expound upon here, because they are more obvious and easier to articulate). In the following sections, we will more closely examine the costs of knapping and their potential implications for the evolution of know-how copying, divided into three main categories: 1) risks and dangers; 2) time, energy, and opportunity costs; and 3) material costs, utility and the problem of waste.

### Risks and Dangers

Potential risks and dangers appear at all stages of the process of making and using stone tools. Transportation of materials and knapping poses dangers in the sense of increased exposure to predators (Caruana, 2020; Hart & Sussman, 2005). During cutting tool *use* (e.g., processing a carcass), there is also a non-negligible risk of cutting oneself and microbial infection. The physiological and psychological effects of stress from interacting with sharp objects are possibly relevant (see Schmidt & Tennie, 2024). In line with the focus of this article, knapping also bears *risks* (e.g., Eren et al., 2020; Gala et al., 2023; Hiscock, 2014; Lycett, 2019; Lycett et al., 2015).

In one of the more explicit accounts of risks, Hiscock (2014) describes how the benefits of cutting edge production should be weighed against the potential risks, such as injuries (e.g., cuts to hands or the eyes) and infections (i.e., due to lack of sanitary practices or antibiotics). Although the degree of risk might vary depending on context, the possibility of injury is a constant during knapping. Because of this, Hiscock (2014) proposes a direct linkage between the risks of knapping and the evolution of social learning, specifically hypothesizing that the “costs/risks were lowered through apprenticeship frameworks for social learning, and apprenticeship learning enhanced fidelity of transmission of *elaborate manufacturing sequences*” (p. 40; emphasis added by the current authors).

Apprenticeship is an especially rich, extended social learning mode (Rogoff, 1990; Sterelny, 2014) and is known from recent knapping contexts (e.g., Papuan adze-makers: Stout, 2002, 2005; Harappan stone-bead maker: Roux et al., 1995). The teaching and supervision of a ‘master’ knapper – according to Hiscock’s account – would reduce the injury risk experienced by the ‘apprentice. Elsewhere, it is argued that specific know-how copying mechanisms like imitation or emulation would be relevant for curbing the injury-related risks of knapping (Lycett et al., 2015, 2016). For example, Gala et al. (2023) draw the conclusion that the “inherently hazardous nature of knapping is more likely to have encouraged the deployment of any social learning capacities possessed by the hominins” (p. 297).

In our view, injury risks *alone* are insufficient for implicating copying of knapping-related know-how, in contrast with the views of other authors (e.g., Gala et al., 2023), who suppose that specific types of (know-how copying) social learning mechanisms and/or delayed learning would have mitigated injury risks. To evaluate this proposed relationship between social learning and risk mitigation, we need to evaluate the following implicit assumptions:

1. The injury costs of knapping were severe enough and frequent enough to noticably reduce an individual’s fitness.
2. Increasing knapper skill will coincide with a reduction in (injury) risk (Hiscock, 2014).
3. Trial-and-error learning, is associated with greater injury risk than learning via know-how copying or via special teaching (Gala et al., 2023; Hiscock, 2014; Lycett, 2019; Lycett et al., 2015).
4. Know-how copying (e.g., imitation) and/or selective teaching (e.g., to delay learning) are better/optimal strategies for (injury) risk mitigation, and therefore would have been selected for.

#### Injury Frequency and Severity

To start, we require a reference for the seriousness and prevalence of knapping injuries. Some, albeit incomplete, insight into this phenomenon is gained from the results of Gala et al. (2023), in which they set out to empirically study knapping injuries by surveying a large sample of contemporary knappers. The study found that injury is not uncommon, even despite the frequent use of protective gear and other safety precautions (e.g., approximately 57% use gloves and approximately 87% use eye protection). Generally speaking, most injuries among contemporary human knappers are rather minor. There were also more severe injuries (i.e., injury to the eye or for which medical attention was sought), but there were very few that might be considered life-threatening. Almost all reported incurring injuries at least infrequently. The injury risk for living human knappers is undeniable, but how was it like in the context of extinct hominins?

First, injury severity could be related to species-specific physiology and behavior, in the sense that, e.g., chimpanzees often survive wounds that would have been fatal for a human (observed by the author CT; see evidence of, e.g., healed cranial fractures in various ape subspecies; Jurmain, 1997; see also Byrne, 2005). Extinct hominins certainly had different bodily affordances and strength than living modern humans (Stout & Semaw, 2006). Differences in wrist morphology, for example, would have implications for injury risk due to the effect it could have on control and accuracy while *freehand* knapping (Kivell et al., 2023; Tocheri et al., 2008; Williams et al., 2014). Freehand knapping is not the only way to knap stones, however, and the postures used by modern replicative knappers are also not the only valid postures for toolmaking (as a reviewer helpfully pointed out, teaching students to knap away from the body instead of against the body can eliminate much of the injury risk; see also Williams et al., 2014 on reduction of leg injuries by instructing knappers not to support cores against their legs). The other panins, bonobos, can employ freehand knapping technique and precision grips, at least to a certain extent (see Cebeiro & Key, 2023; Toth et al., 2006), but also use alternative strategies (like projectile technique), which may impose less risk than the culturally-derived form of body-supported freehand knapping that modern replicative knappers have come to prefer. Recognizing that early toolmaking hominins indeed flexibly applied different techniques across different contexts (e.g., Delagnes et al., 2023; Toth & Schick, 2018), they may have simply reduced injury risk by employing different posture and/or gestures than that typical of living knappers (e.g., Rein et al., 2014; Snyder et al., 2022).

Second, injury risk is relative to the technological strategies of the stone tool industry. All else being equal, later technologies plausibly carry greater risk per core than earlier stone tools if only because the former demand longer operational chains to produce (e.g., Muller et al., 2022; Stout et al., 2021) or because of the strength and precision requirements (Toth & Schick, 2019; however). Hominins may have becoming increasingly reliant on making stone tools requiring complex reduction sequences and greater precision (and subsequently more frequent use of freehand knapping). But in the case of achieving greater control and accuracy, biological adaptations, such as increased wrist extension (see Williams et al., 2014), could have also reduced the injury risk involved (i.e., without needing to implicate adaptations related to know-how copying).

Finally, if the earliest toolmaking hominins were *not habitual* toolmakers (see Karakostis, 2023; Shea, 2017; Toth, 1985), overall risks (and consequently, selective pressure) would be less significant. More generally, it is useful at this point to separate out two factors contributing to the overall risk profile associated with a given technology– whether stone or otherwise. Specifically, Bamforth and Bleed (1997), following Torrence (1983, 1989) distinguish between the cost of a technological failure, on the one hand, and the probability of such a failure, on the other. Sometimes, the cost of a technological failure is major (e.g., you starve); at others, it is minor (e.g., you merely go to bed hungry). The magnitude of this cost is important as it shapes the level of failure-probability that is tolerable; a high chance of failure may be acceptable if the cost of failure is low, while even a low chance of failure may be unacceptable if the cost is high. In a similar way, one might argue that so long as the costs of a knapping mishap remained low, even knapping strategies with a high error-rate may still have proven tolerable. A high chance of injury might have been (evolutionarily) inconsequential if those injuries tended to be quite minor. If so, then the argument from risk reduction for high-fidelity transmission of knapping skill via know-how copying is undermined.

#### Skill Level and Risk Reduction

Based on self-report, a majority of living human knappers (approximately 75%) reported injuring themselves more often in the past (Gala et al., 2023). At face value, this gives the impression that increased practice and skill level does indeed lead to a reduction in knapping-related injury risk. But given that this is based on self-report, we cannot precisely map out the relationship between, e.g., practice time and injury frequency or skill level and injury severity. Longitudinal data collection would provide more accurate, higher resolution data on potential changes in injury risks during skill acquisition, although targeted investigation of knapping injuries has ethical ramifications.

Based on the cross-sectional dataset of Gala et al. (2023), experts also incur injuries during knapping. The risks of knapping can never be completely eliminated (see Hiscock, 2014): they largely stem from *uncontrollable aspects* of tool manufacture. This naturally and empirically limits the extent to which know-how copying can possibly drive down the associated risks.

#### New and/or Enhanced Types of Social Learning

The main proposed strategies for reducing the risks of knapping are the adoption and/or employment of know-how copying, teaching of know-how, or even apprenticeships, because these are theorized to result in less risk for knapping novices than, e.g., trial-and-error learning (Gala et al., 2023; Hiscock, 2014; Lycett, 2019; Lycett et al., 2015). At the outset, it should be pointed out that – to our knowledge – there is no published data that measures risk frequency and severity in different learning conditions which would allow the assessment of such hypotheses on the relationship of learning type and (injury) risk. Likewise, we are unaware of any study examining the specific causal variables related to knapping-incurred injury (much less how these variables interact with the type of learning in play). Intuitively, one would expect the relevant causal variables to include, e.g., visual acuity, hand–eye coordination, control over the hammerstone/core, control over striking velocity, etc., but systematic empirical data on this issue would be clearly preferable. However, in our view, even this intuitive list of factors is sufficient to challenge the idea that knapping risks selected for the employment of know-how copying. Different learning strategies may well differ in their injury risks, but there are other mitigation strategies that would have been within the adaptive reach of prehistoric hominins.

First, there is reason to think that it is safer for inexperienced knappers to work with whatever production behaviors feel most natural for them (cf. Eren et al., 2020), as opposed to trying (and failing) to reproduce specific techniques of older and/or expert knappers (Rein et al., 2014; see also Snyder et al., 2022), which may well be demanding of things such as strike accuracy and control of arm velocity. Furthermore, modern novices who do not yet have the requisite motor coordination and/or strength to produce *observed* knapping know-how of expert models in fact tend to innovate different techniques – ones that are conceivably less demanding or taxing; e.g., bipolar technique(s) (human adults: Pargeter et al., 2023; human children: Ferguson, 2003; Sternke & Sorensen, 2009) or projectile and anvil-based techniques (bonobos: Eren et al., 2020; Toth et al., 2006) – and different knapping gestures from the ones they observe (Rein et al., 2014). Even if we were to assume the presence of know-how copying, such strategies on the part of novices may well have compensated for potential risks of knapping. In other words, any know-how copying abilities they possessed need not even have been employed.

Second, trial-and-error learning tends to be taken as a main foil to know-how copying (cf. Lycett, 2019; Lycett et al., 2015), but this characterization misrepresents the diversity of individual learning types and their evolutionary role in hominin cognition (see, e.g., Eteson et al., 2024; indeed, individual learning types would be similarly diverse to social learning types; Table 2, 3). Regardless of the debated relationship of social learning with knapping, the role of hands-on practice is undeniable. One cannot simply learn how to make stone tools by observation alone, even where there is scaffolding and/or explicit teaching of know-how involved (e.g., Harlacker, 2003; Pargeter et al., 2019, 2020; Rein et al., 2014; Snyder et al., 2022; Sterelny, 2014; Stout & Semaw, 2006; Stout et al., 2011, 2014). This suggests, in parallel to any continuum of social learning strategies, there would be a continuum of individual learning strategies (with trial-and-error learning on one end and deliberate practice on the other). Changes in how hominins individually learned could have led to a reduction in the risks of knapping (perhaps even a ‘good’ individual learner is better than a ‘poor’ know-how copier).

#### Delaying Learning

The idea that the risks of knapping were managed by delaying childrens’ exposure to the activity – in contrast to the learning model of Eren et al. (2016) – infers that hominin adults actively controlled the learning activities of their young. That is possible in principle. As Gala et al. (2023) cite, there is a reported tendency of mother chimpanzees to ant-dip more at trail sites than at nest sites while foraging with their young (Lonsdorf, 2013). Because trail sites expose the ant-dippers to less risk than nests, the behavior of the mother chimpanzees could equate to them actively delaying the exposure of their young to the riskier of the two sites. To argue for delayed learning mitigation strategies, Gala et al. (2023) also invoke ethnographic accounts of Konso hide workers and adze makers in Irian Jaya (Stout, 2005; Weedman Arthur, 2010), for whom toolmaking praxis begins in the teenage years.

We have serious reservations about this interpretation, as primatological and ethnographic evidence can also be invoked in contradiction of this logic. In the case of apes, learning delays (if intentional) may rather exist to facilitate parenting. Even further, delays need not imply any know-how copying. For example, a delay may result from needing sufficient time to *individually* master the sensorimotor demands of an activity. Apes engage in many risky behaviors, like climbing (e.g., Teleki, 1973), and ‘unpleasant’ activities, like ant-dipping (Humle et al., 2009) – without much caregiver interference (other than perhaps, teaching of know-*when*-*not* and know-*where*-*not*). In general, teaching any type of knowledge in apes is very rare (Moore & Tennie, 2015) – and the few cases are debated – despite many behaviors involving risks. Where it does occur, teaching takes a form that is quite distinct from human stone toolmaking apprenticeships (see Table 3; Stout, 2005).

Even more telling, in our view, is ethnographic evidence from forager societies. Far from preventing children from dangerous exploratory learning, human children are often (implicitly) encouraged to *individually* engage in such behavior in many such societies (see, e.g., Lancy, 1996). This tendency strongly contrasts with the norm in WEIRD (Western, Educated, Industrial, Rich, Democratic; Henrich et al., 2010) cultures. To give one example, Little (2011) found that Asabano children in Papua New Guinea are often left unsupervised, venturing into dangerous areas of the jungle, carrying and using knives and razorblades, and starting fires. Parents not only do not intervene in this potentially injurious behavior, but they even provide the children with such dangerous objects. Contrary to the logic of the delayed-acquisition hypothesis, forager parenting tends to be hands-off, with teaching of children by adults being only a minor component of learning in foraging societies and autonomous learning being more standard (see Boyette & Hewlett, 2017; Lew-Levy et al., 2020; also Sterelny, 2022 on late Pleistocene foragers). As for the ethnographic examples cited by Gala et al. (2023), knapping, like in Papua (Stout, 2002, 2005), is bound to apprenticeship systems that are non-parsimonious for late Pliocene and early Pleistocene toolmakers (requires unvalidated assumptions for the teaching of know-how, language, and modern human-like demography and sociality). Learning delays in these societies may also relate more with raw material conservation rather than mitigating risk to children (Ferguson, 2003; Stout, 2002, 2005; see below).

Finally, it should be noted that delayed learning mitigation strategies would themselves impose costs (on learners and teachers), at least to the extent that knapping skill is important to fitness (cf. Shea, 2017; Toth, 1985). By delaying opportunities for hands-on practice, the acquisition of skilful and *productive* toolmaking would also be delayed. Especially if injuries were only very rarely life-threatening, there may have simply been too little evolutionary pressure to delay learning anyway (i.e., delayed learning would have higher costs than actual benefits). In fact, instead of delaying learning completely, hominins could have employed teaching of know-*w* to gradually expose their young to the more dangerous steps of behavioral procedures (see Table 3 and consider the example of teaching via know-what in meerkats; Thornton & McAuliffe, 2006). For example, in the Early Acheulean, adult hominins could have manufactured the large flake blanks (which is a dangerous step and requires enormous strength; Toth & Schick, 2019) and allowed younger individuals to knap some of the resultant blanks and discard pieces (compared with embedded learning strategy: Ferguson, 2003).

### Time, Energy, and Opportunity Costs

Next, we turn our attention to time, energy, and opportunity costs. The argument goes something like this: where a skill is important to survival, and takes non-trivial amounts of time and energy to acquire (e.g., Pargeter et al., 2019, 2020; see also the ‘transmission time investment model’ of Kovach & Gill, 2023), selection will favor the ability to copy the corresponding know-how, as opposed to individually (re-)inventing that know-how for themselves (see Hiscock, 2014; Lycett et al., 2016). Per our understanding, this argument rests on the following series of assumptions:

1. Early toolmaking (and learning how) required investment in terms of time and energy.
2. Hominins who learned expediently and efficiently would have gained fitness benefits, while slow-learning hominins would have had a disadvantage. In more extreme terms, hominins *needed* to learn to knap quickly.
3. Learning that is demonstrably quick and efficient *in living modern humans* (i.e., know-how copying) was also comparatively quick and efficient in hominins.
4. The overall cost–benefit relationship would have resulted in selection for increasingly advanced know-how copying abilities (for ever increasing expediency and efficiency).

#### Time and Energy Investment and Missed Opportunities

The plausibility of this cost-based argument depends on early stone tool manufacture indeed having significant time and energy costs. In isolation, a single cutting flake can be produced in a negligible amount of time. So proportionally, most time and energy costs would be incurred during material selection and transport (e.g., Braun et al., 2008, 2009; Favreau, 2023; Goldman-Neuman & Hovers, 2012; Reeves et al., 2021, 2023; Stout et al., 2010; Toth, 1985; Toth & Schick, 2018) or while *learning to* knap (e.g., Bril et al., 2010, 2012; Pargeter et al., 2019, 2020, 2023, 2024; see also Eteson et al., 2024), the latter of which we discuss here (for now, excluding the energy required to have and maintain the ‘appropriate’ knapper physiology; see Pargeter et al., 2019; Stout et al., 2015, 2019; cf. Haslam et al., 2009).

Living human knappers need an extended period of time to master knapping of later stone tools, such as Late Acheulean toolmaking skills, even when provided ample opportunities for know-how copying (including explicit teaching, language, and social prestige-related motivation; Pargeter et al., 2019, 2020; Stout et al., 2014). Especially with such extended learning, the time spent on collecting materials and practicing toolmaking could have otherwise been spent on other daily functions, such as reproduction or non-knapping-related foraging. For Oldowan and Early Acheulean tools, neither of which reach the same degree of technical complexity and cognitive demands as Late Acheulean tools (Muller et al., 2022; Stout et al., 2008): how much time and energy was needed to learn to make *these* tools?

To begin, a distinction must be drawn between *mastery* (reaching near peak skill and efficiency) and *adequacy* (an ability to produce a functional outcome). An ‘adequate’ (adequacy being in the eye of the beholder, i.e., the lithicist) toolmaker would be competent enough to produce *minimally* usable cutting tools. It seemingly does not take contemporary humans very long at all to acquire basic Oldowan knapping principles, even in the absence of know-how models (see Snyder et al., 2022). On the other end, the timing of toolmaking *mastery* (in both humans and hominins) remains unknown territory. There are living knappers that have attained skill comparable to that of the most skilled Oldowan knappers, but no study *thus far*, regardless of the learning conditions, has documented how a person can go from being naive to having attained the skill level indicated at sites like Gona, Ethiopia (cf. Pargeter et al., 2023; Snyder et al., 2022; Stout & Semaw, 2006; Stout et al., 2009; Toth & Schick, 2009).

It has been supposed that Late Acheulean and even Oldowan toolmakers engaged in *deliberate* knapping practice (i.e., an activity separated from foraging functions) as a necessary part of skill acquisition (Pargeter et al., 2019, 2020; Stout et al., 2015, 2019). The presence of such a learning strategy is increasingly presumptive the further one goes back in time, and such deliberate practice would itself likely be the end-product of selection on earlier types of individual learning (cf. Eteson et al., 2024; Stout et al., 2019; consider also a feedback loop between social and individual learning types: van Schaik & Burkart, 2011), which we find more parsimonious for the Oldowan and Early Acheulean (Snyder & Tennie, 2023; Snyder et al., 2022; Tennie, 2023; Tennie et al., 2016, 2017). Instead of engaging in a frequent, *dedicated* knapping practice or even frequent knapping generally speaking (see Shea, 2017), Oldowan and Early Acheulean toolmakers may well have had a more ‘relaxed’ make-as-you-go approach to knapping, involving on-the-spot flake production in the service of extractive foraging (resulting in a kind of ‘learning by doing’ – similar to that of apes). Such an embedded learning strategy would absorb much of the time and opportunity costs that would otherwise be accrued, especially with explicit forms of dedicated practice. (As pointed out to us by one of the reviewers, these different types of approaches to knapping plausibly predict different archeological traces, e.g., as regards the amount and type of stone debitage produced at a knapping site. However, we leave further discussion of this important issue for a future work.)

Not only do we not have an estimate for learning time under ideal conditions for WEIRD humans (see Pargeter et al., 2020, 2023) or for extinct hominins (regardless of whether they had dedicated practice or long-term fluid, embedded learning; Snyder & Tennie, 2023; Snyder et al., 2022), but we also cannot compare the full trajectories of different learning conditions. This last point is especially relevant when teasing apart the issue of *efficiency* and *expediency* in learning to knap.

#### Expediency and Efficiency

The next question is: did hominins acquire knapping skills quickly and was there selective pressure to do so more quickly? This matter of efficient and/or expedient learning of knapping know-how is core to the debate about know-how copying in early prehistory.

It is telling in our view that archaeologists have identified little evidence of *practice* pieces (i.e., exclusively identifiable as products of novice toolmakers) in Oldowan assemblages (see, e.g., Braun et al., 2019; Delagnes & Roche, 2005; Stout & Semaw, 2006). And yet, in all tested learning scenarios, modern human novices have produced artifacts that match neither modern human experts’ products nor archaeological material (e.g., Pargeter et al., 2021; Snyder et al., 2022; Stout & Semaw, 2006; Stout et al., 2009). There are multiple ways to interpret this. Perhaps the evidence simply did not survive to the present (e.g., Dibble et al., 2017). Or, learning how to knap may have simply been fast among these hominins. It may be, given the general lack of sophistication of Oldowan artifacts, that we lack reliable methods for recognizing the products of less versus more skilled individuals in the archaeological record. If we compare this with the primate archaeological record, artifactual and (between and within) assemblage variation seems to relate more with ecological and raw material factors than learning factors, meaning there is little to show as far as ‘practice pieces’ (e.g., Arroyo et al., 2021; Falótico et al., 2019; Reeves et al., 2024), and yet actualistic research informs us that skill acquisition of tool use can be rather protracted (see, e.g., work on stick tool use acquisition in wild chimpanzees; Malherbe et al., 2024). From this, we might hypothesize that learning in early hominins was also relatively protracted, but did it *need* to become quicker?

The need to learn more quickly, in the evolutionary sense, requires the inference that fast learning imbues fitness benefits and slow learning leads to a (significant) selective disadvantage. But in this case, we would have to assume that hominins *needed to knap* at all in order to survive. For one, the archaeological record does not provide us with data that would demonstrate that all hominin populations were knappers, that late Pliocene and early Pleistocene hominins were habitual knappers, or let alone, that these hominins depended on knapping for survival (see Shea, 2017; Toth, 1985). In fact, not only do primate models (see differential distribution of tool use behaviors across ape populations: e.g., Acerbi et al., 2022; Whiten et al., 1999; and studies on stone tool use and toolmaking in primates: Bandini & Tennie, 2023; Bandini et al., 2021b; Motes-Rodrigo et al., 2022) suggest that stone toolmaking likely did not appear in *all* late Pliocene and early Pleistocene hominin groups (see also Dusseldorp & Lombard, 2021), they were also not dependent on stone tools for their survival needs (considering foraging activities *sans* knapped stone tools; Bandini et al., 2022; Hayden, 2008; Hovers, 2012; Pargeter et al., 2019; Schick & Toth, 2006; see also Toth, 1985). There are also other adaptations to consider. For example, if certain evidence regarding evolution away from an ‘ape’ model and towards a more ‘human’ model of life history and food sharing practices in early *Homo* (see, e.g., Alger et al., 2023; Hrdy, 2009; Opie & Power, 2008; Sterelny, 2022) are to be believed, then young and/or novice toolmaking hominins were supported and, e.g., calorically subsidized by other, perhaps more advanced group members.

### Material Costs, Utility, and the Problem of Waste

Finally, we turn to a third line of cost-based thinking, this one revolving around *raw material costs*. To start, the procurement of raw materials requires (again) the expense of time and energy (including demands on attention and the body), related to walking, detecting materials, selection of appropriate (or even higher-quality) materials, and transportation, going significantly beyond what is observed in non-human primate tool interactions today (e.g., Braun et al., 2008; Goldman-Neuman & Hovers, 2012; Reeves et al., 2023; Toth & Schick, 2018; Wynn et al., 2011). The raw materials for making tools are not inexhaustible (Ferguson, 2003). As more local materials are used up (or if materials of different or better qualities are sought for), knappers would – eventually – have to import further materials from farther away sites (Ferguson, 2003; Hiscock, 2014; Shelley, 1990). There are then the risks and time and opportunity costs of searching for new sources. Material costs are an unavoidable part of knapping (it is, in essence, a reductive method; Ferguson, 2003), and they also vary during an individual’s learning curve. The set of assumptions connecting raw material costs and conservation with evolving capacities of know-how copying would goes as follows:

1. Raw material costs due to inefficiency would have been considerably greater during learning than during later stages of toolmaking mastery.
2. Raw material costs due to waste, i.e., the production of ‘bad’ or unusable tools, would also have been greater during learning.
3. Know-how copying provided a selective advantage, because know-copying types would have relatively increased the efficiency of learners in exploiting stone volumes, while also reducing the rate of mistakes leading to ‘bad’ tools.

#### Efficient Management of Raw Material Volume

The raw materials that are collected may be of a low quality, which could result in the expenditure of proportionally even more energy to knap or require a higher volume of materials to meet needs (e.g., Favreau, 2023; Goldman-Neuman & Hovers, 2012; Reeves et al., 2021). Just as well, unskilled or inefficient knapping may result in the ‘wasting’ of materials that will incur further costs (e.g., Ferguson, 2003). Because novices are not able to immediately achieve the reduction efficiency of experts (i.e., novices do not optimize their exploitation of the initial stone volume, with optimization related to, e.g., cutting edge production or amount of flakes), this also imposes costs in terms of the relative amount of required material and wasted volume (see Ferguson, 2003; Hiscock, 2015; Lombao et al., 2017; Pargeter et al., 2021; Shelley, 1990; Stout & Semaw, 2006; Stout et al., 2009).

The true disadvantage of inefficient or wasteful use of raw materials, however, is quite contextual. It depends on the preciousness of the raw materials, related e.g., to the availability and accessibility of the materials, and consequently, the time and energy costs of acquiring them. Raw materials that are locally available with little need for pre-processing, for example, would incur little risk or cost. As mentioned above, the habituality of toolmaking and use (e.g., Shea, 2017) is a consideration. Hominins picking up loose stone nodules and expediently flaking them when needed (Isaac, 1984; Toth, 1985) would not be under the same (selective) pressure to avoid ‘waste’ as hominins that are *dependent* upon knapping stone tools for their survival.

#### ‘Bad’ Tools

The interpreted quality of products will also vary, and it may be wasteful when the knapping products deviate too much from the supposed ideal (cf. Davidson & Noble, 1993; Ferguson, 2003; Hiscock, 2014; Lycett, 2019; Lycett et al., 2015; Nishiaki, 2019; Pargeter et al., 2019, 2020). Assuming that there is a normative or cultural ideal or a specific utility for the produced tool, costs are incurred when knapping products are imperfect or unusable, i.e., opportunity costs of lost material that otherwise could have been used for something else.

The interpretation of the knapper’s goals is key here (see Davidson & Noble, 1993). As far as earlier knapped stone tools (especially for the Oldowan and perhaps also the Early Acheulean) are concerned, there seems to be a lack of design plan (Moore & Perston, 2016; Snyder et al., 2022; Toth, 1985). The crux of what is a ‘good’ versus a ‘bad’ tool therefore lies not on any cultural ideal but on utility: what constitutes a functionally adequate artifact? In this case, we are too often guided by a sense of flintknapper’s conceit when studying tool utility (e.g., Eren et al., 2016). In reality, the morphology of these tools does little to indicate the actualized role and use in hominin activities (e.g., Hiscock, 2014, 2015). Ethnographic evidence of stone tools, including superficially Oldowan-like ones, made by Aboriginal Australians (Binford & O’Connell, 1984; Hayden, 2008, 2015) suggests that not only do ‘bad’ tools (as far as we might interpret from the perspective of a contemporary WEIRD archaeologists; see Killin & Pain, 2022 for the WEIRDness of cognitive archaeology; see also Bandini et al., 2022; Hayden, 2008) have a wide breadth of utility (sometimes simply how well one might grip a tool can take priority; e.g., Holdaway & Douglass, 2011), but also the (by)products of knapping just a bit of stone can be used for a wide range of tasks. Bulk production of flakes can be a very effective strategy for useful cutting-edge production, with utility of each candidate tool judged on a case-by-case basis for whichever task the individual wants to complete at a given time (see Holdaway & Douglass, 2011; Shea, 2017). This would include not just cutting-based extractive foraging tasks, but as, e.g., weapons (even simple projectiles) against out-group members or predators (see Toth & Schick, 2009).

A knapper who is ‘bad’ or ‘wasteful’ cannot be a priori assumed to have suffered so severely from their lack of skill (see again Bamforth & Bleed, 1997). Even crude or dull implements made by hominin novices could have sufficed for achieving (some of) their goals, which undermines the assumption that there would be a strong selective pressure (to better or more efficiently learn how) to make ‘good’ tools. And again, we cannot disregard the possibility that ‘bad’ toolmakers received caloric ‘subsidies’.

#### Cost Mitigation Strategies

Assuming *for the moment* that these costs were important enough to impact the fitness of the earliest knappers (which is, again, not a given), the expectation is that these hominins would have developed strategies to reduce and/or minimize these costs.

One possibility is that employing know-how copying would have led to more efficient use of raw materials over an extended period of time relative to other learning strategies that would include individual (e.g., trial-and-error) learning, appropriate social scaffolding (see Sterelny, 2022), know-*w* social learning, and know-how triggering. Our earlier point about the tendency of inexperienced knappers to employ production behaviors that are most natural *for them *(at least at the time), as opposed to attempting to reproduce the behaviors of older/more expert knappers, is again relevant here. It is not hard to imagine inexperienced individuals actually going through *more* raw materials as they attempt to match a technique that is expressed by the expert individual they have observed but that is beyond their current skill level (see, e.g., Pargeter et al., 2023; Rein et al., 2014). Instead, it may be better to opt for techniques that make more out of less (like bipolar technique; cf. Caruana, 2020; Diez-Martín et al., 2011; B. Morgan et al., 2015a, 2015b). And again, it is unclear whether know-how copying would have even been a more efficient way to learn how to knap (see above).

It is possible that (the development of) other learning strategies such as know-*w* teaching (e.g., kinds of basic scaffolding) can also reduce the disproportionate material costs incurred by learners. This would include raw material sharing or re-use of cores and tools (for a primate example of this phenomenon see Fragaszy et al., 2013; see also description of embedded learning of knapping by Ferguson, 2003). Again, the notion of sharing or at least social facilitation (tied also to life history and social organization; see Sterelny, 2022) as traits for selection provides an alternative evolutionary pathway that need not implicate know-how copying or selection thereon in the earliest portion of prehistory.

## Evaluating the Models

**Fig. 1:**
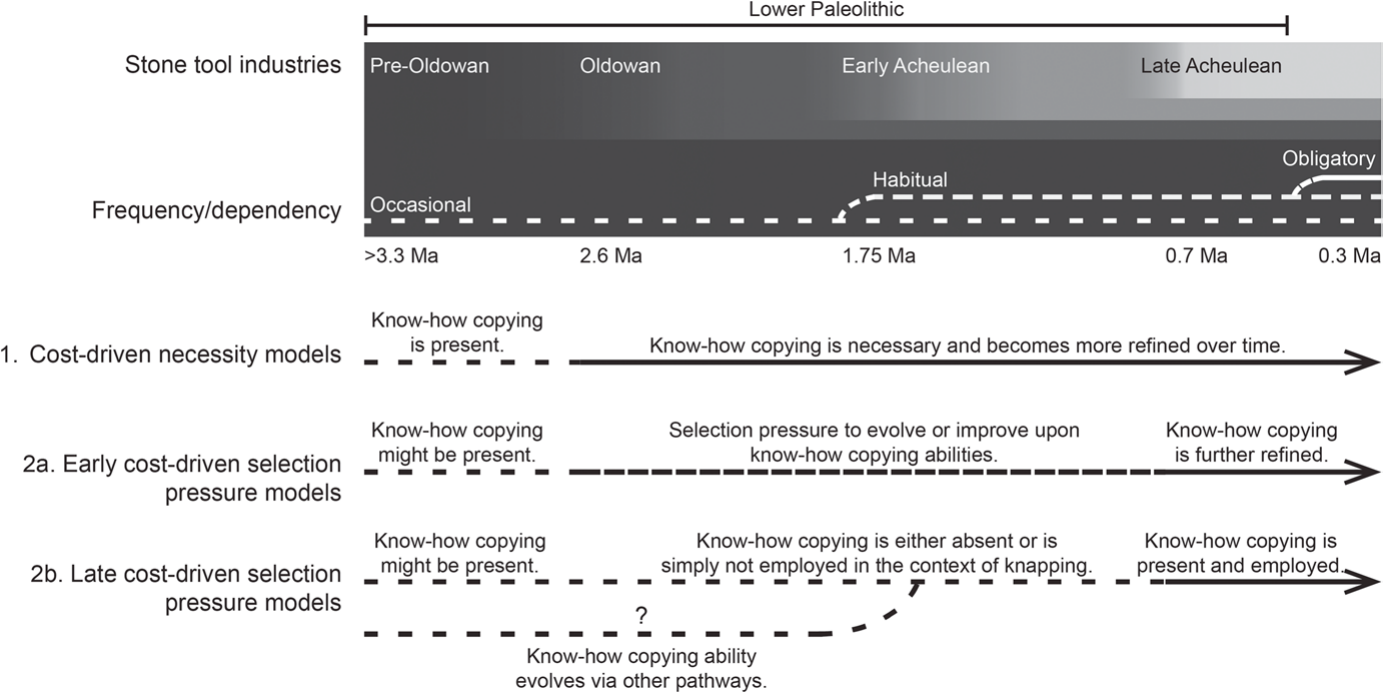
Timeline showing the respective stone tool industries, the estimated frequency of/dependency on stone toolmaking (Shea, 2017), and a comparison of the main cost-driven estimation models

Certain shared aspects of cost-driven estimation models are uncontroversial, e.g., that knapping is costly or that there was some relationship between knapping and social learning. Other aspects of these models are less clear-cut. Important factors include the (relative) magnitude of the knapping costs, the nature of the relationship between knapping costs and social learning, and separate factors, like alternative mitigating processes/strategies and other factors that can influence the relative fitness improvements related to social learning itself (Fig. 1). The link between knapping costs and social learning is simply not as easy to identify as has been previously assumed in the archaeological literature. Based on the preceding sections, we would draw the following conclusions about the different cost-driven estimation models.

We begin with what we consider the most extreme case: *cost-driven necessity models* (Fig. 1). We are not convinced by arguments that posit a link between the costs of knapping and a necessity for know-how copying. Indeed, if this kind of link were to be applied to other types of behaviors, then the costs of a behavior for a wide-range of animals, living or extinct, could potentially be used to invoke a necessity for know-how copying. And yet, know-how copying has proven rare in the animal kingdom. For example, sea turtle hatchlings must make it into the water, or else succumb to a veritable onslaught of predators. And yet, over many millions of years, these obvious steep costs and risks (and lost fitness) have not led to the mitigation of these risks via mother turtles demonstrating (and/or teaching) hatchlings *how* to avoid predation. The same is true for wood ducklings jumping out of their natal trees and infant chimpanzees climbing trees (etc.). Moreover – and perhaps most germane of all to the current debate – the same can be true even for living modern humans, where the costs imposed by certain, especially dangerous, behaviors go unmitigated by know-how copying, even when deployment of know-how copying *could* potentially significantly reduce the risks involved. Dangerous individual experimentation with technology (e.g., knives) and natural resources (e.g., fire) may not only be permitted, but even encouraged (e.g., Lancy, 1996; Little, 2011; autonomous learning rather than adult–child teaching being more important in foraging societies; Boyette & Hewlett, 2017; Lew-Levy et al., 2020). Viewed from this perspective, the logic that costliness of a behavior, including knapping, implies the early evolution of a *need* to copy know-how is not persuasive.

Turn now to the *cost-driven selection pressure models*. To properly evaluate this cluster of models, it is necessary to consider each subcategory (Fig. 1). In vague terms, the idea that the costs of knapping would have created a selection gradient that led to (subsequently improved) abilities for know-how copying *at some point in time in our lineage* is an attractive one. But framed this way, it also does not say anything truly meaningful about human cultural and cognitive evolution. A number of important questions are left unanswered by such a vague statement, including those related to the approximate timeframe in which know-how copying evolved, when know-how copying became necessary to transmit certain knapping skills, under what selection pressure(s) it evolved, and under what selection pressure(s) it specifically became involved in the transmission of knapping know-how. When split into these parts, it becomes clear that the disagreements among evolutionary archaeologists are very much a ‘chicken-and-egg’ debate, where the same relationship is being described but the elements are sorted into distinct timelines. Put simply, our ‘chicken’-proponents use *early cost-driven selection pressure models* (see 2a)*,* and our ‘egg’-proponents use *late cost-driven selection pressure models* (see 2b). For full clarity, however, we are not suggesting that there was a specific time point in the past where fully-honed (contemporary-) human-like abilities of know-how copying popped into existence. Not only is there (likely) variability of this ability today, but such a saltatorial approach would require highly special circumstances, unlikely to have been present in the time of the earliest toolmakers.

As the name suggests, *early* cost-driven selection pressure models assume that human-like know-how copying was either already present *in the early archaeological record* or that it would have emerged within that timespan due to the demand to quickly and effectively learn to knap and mitigate costs involved (see studies such as Cataldo et al., 2018; Lombao et al., 2017; T. Morgan et al., 2015a, 2015b; Putt et al., 2014; Pargeter et al., 2019, 2020, 2023; Shipton, 2020). Even where authors claim to be concerned only with a selective gradient for the copying of know-how and not the precise timing of the advent of know-how copying (e.g., Pargeter et al., 2023), there is still an *a priori* assumption of *some*, if simpler and/or less powerful, know-how copying ability present before and during the Oldowan and Early Acheulean in their models of cognitive evolution. They do not agree (cf. Pargeter et al., 2023; Wilson et al., 2023), for example, with our stance that the earliest knapped stone tools did not require know-how copying and were instead based on know-*w* copying, know-how triggering and individual contributions (Snyder & Tennie, 2023; Snyder et al., 2022; Tennie, 2023; Tennie et al., 2016, 2017). This rejection of the ‘zone of latents solutions’ account *de facto *indicates an assumption of the early presence of know-how copying of *some* kind(s) in the thinking of these authors.

Alternatively, one might advocate that – though there still would be a selection gradient leading towards the cultural transmission of knapping know-how at some point in time (Fig. 1) – know-how copying was likely *not* present during the Oldowan and Early Acheulean. The starting point of the selection gradient would therefore be a substrate including know-*w* social learning, know-how triggering and individual learning (because Oldowan knapping in-of-itself does not pre-suppose know-how copying; Snyder & Tennie, 2023; Snyder et al., 2022; Tennie, 2023; Tennie et al., 2016, 2017; see also Eteson et al., 2024 on a selection gradient related to individual practice). Again, the involvement of such social learning *sensu lato* is uncontroversial: social learning of some kind was expressed by those early hominin populations (contra erroneous portrayal of the debate in, e.g., Gala et al., 2023; Shipton, 2020). We assume that even weak, gradually evolving know-how copying abilities would have been detectable in some way, and based on our trait-based estimation of these abilities, we find no strong evidence to assume know-how copying was involved *in stone toolmaking* during the Oldowan and Early Acheulean (see Snyder & Tennie, 2023; Snyder et al., 2022; Tennie, 2023; Tennie et al., 2016, 2017). From this starting point, we could then ask the question of why know-how copying initially emerged. It is possible that the evolution of know-how copying was actually driven by an entirely different set of factors; for example, factors relating to some other type(s) of tool use that is invisible in the record or factors relating to enhanced communication (e.g., an expanded role for gestural communication in early *Homo*), among other possibilities (e.g., Boyd et al., 2011; Fragaszy et al., 2024; Henrich, 2015; Planer, 2017, 2023; Reindl et al., 2016; Richerson & Boyd, 2000; Rolian & Carvalho, 2017; Timmermann et al., 2024; van Schaik & Burkart, 2011). Yet another, distinct pathway for the emergence of know-how copying has also been put forth: via an incipient evolutionary transition in individuality that was enabled by ape-like population structures in conjunction with the variedness of their traditions (Andersson & Tennie, 2023). With this in mind, (the costs of) knapping may not have been the main or sole factor responsible for the initial appearance of know-how copying, if it was even a driver at all. Once hominins had evolved know-how copying abilities – regardless of the exact initial pathway – these abilities could have then been selected for (i.e., for the mitigation of knapping costs and other drivers) and evolved upon, at least leading up to the point in time when hominins might have become obligate toolmakers, thus experiencing intensifying evolutionary demands to reduce costs and optimize the ‘reproduction’ of know-how (cf. Pargeter et al., 2019, 2020, 2023; Shea, 2017). In other words, if knapping and its costs were an important factor in the emergence and/or subsequent evolution of know-how copying, the material evidence to indicate this process only shows up in the Late Acheulean, the earliest (even if some or all of the puzzle pieces existed before then; see e.g., Paige & Perreault, 2024; Planer et al., 2025; Tennie et al., 2017; van Schaik et al., 2019).

None of the arguments advocating a direct link between know-how copying and the manufacture of early stone tools even approach being bulletproof, nor can any proposed model accurately delineate the actual evolutionary relationship that might have existed between know-copying and knapping costs (again, we are dealing with extensive time periods and broad strokes, not any one particular moment in time). The notion that know-how copying might have been *beneficial* to learners if it had been present and if it had been used, given, e.g., the risks of stone tool manufacture (or the time and energy costs of skill acquisition, or the material costs), is plausible. But just showing the hypothetical benefits does not demonstrate the evolvability of know-how copying under the relevant ecological conditions. At the very least, one would need to show that these benefits must have outweighed the costs inherent in evolving (developing) and deploying the novel social learning abilities (cf. Hiscock, 2014; Lycett, 2015, 2019; Lycett et al., 2015). These costs might have been substantial, and the benefits associated with know-how copying might not actually have been all that great. Indeed, the net benefits of know-how copying would have had to sufficiently exceed those of other learning pathways, in order for create (more than gradual) selection for the requisite cognitive traits (cf. Lycett, 2015). It may well be that, not only were other social learning mechanisms besides know-how copying sufficient for the know-how in question to appear (Snyder & Tennie, 2023; Snyder et al., 2022; Tennie, 2023; Tennie et al., 2016, 2017), but also know-*w* social learning (or even other mitigating factors like the evolution of new life history traits, among others) could also still provide benefits for mitigating costs, as opposed to just pure individual learning (*contra* Gala et al., 2023; Hiscock, 2014; Lycett, 2015, 2019; though see Eteson et al., 2024). Just as well, as examples from modern humans (sharp object play among children; Lancy, 1996; Little, 2011) remind us – such cost–benefit analyses are not logically binding in pointing to know-how copying. Humans today do not (always) use teaching via know-how copying in situations where the costs are potentially very high. And finally, the link between costs and know-how copying is highly doubtful, because the relative rarity of know-how copying in the animal kingdom (despite no rarity of costly – and even extremely costly – behaviour!), which plausibly suggests that there are real hurdles associated with evolving this particular ability (see Andersson & Tennie, 2023). Especially, our closest relatives, apes, do not copy know-how to anywhere the degree necessary (any maybe not at all), yet they should have a prerequisite substrate of cognitive abilities fairly similar to that of extinct hominins and engage in similarly dangerous behaviors (the costs of termite fishing might just as well be proposed as a driver of know-how copying abilities in apes and/or hominins).

## Conclusion

The intentional production of sharp edges on stone is seen as a major development in the evolutionary history of the human lineage (Harmand et al., 2015; Schick & Toth, 1994; Shea, 2011, 2017; Toth & Schick, 2018). The production of these sharp edges brought with it the expansion into a new ecological niche, so that hominins could exploit new resources and make better use of old resources (e.g., Caruana, 2020; Davidson & McGrew, 2005; Iovita et al., 2021; Snyder et al., 2022; Wynn et al., 2011).

Just as knapping had its benefits, it also brought with it particular costs, some of which are not present in behaviors like nut-cracking (see, e.g., Bril et al., 2010, 2015; see Stout, 2005 on differences in technological skill acquisition between chimpanzees and living human stone toolmaking societies). The costs of knapping are evident, and the costliness of knapping has been implicated by a number of theorists in an evolutionary process that would have resulted in human-like abilities for know-how copying, abilities distinct from the types of social learning that predominantly underline cultural behavior in apes (see Bandini et al., 2020; Tennie et al., 2009, 2020). Though hominins were surely social learners, and very likely social learning was involved in stone toolmaking behavior, we do not see any irrefutable evidence for any type of know-copying following a trait-based approach to studying the available evidence (Snyder & Tennie, 2023; Snyder et al., 2022; Tennie, 2023; Tennie et al., 2016, 2017).

**Fig. 2:**
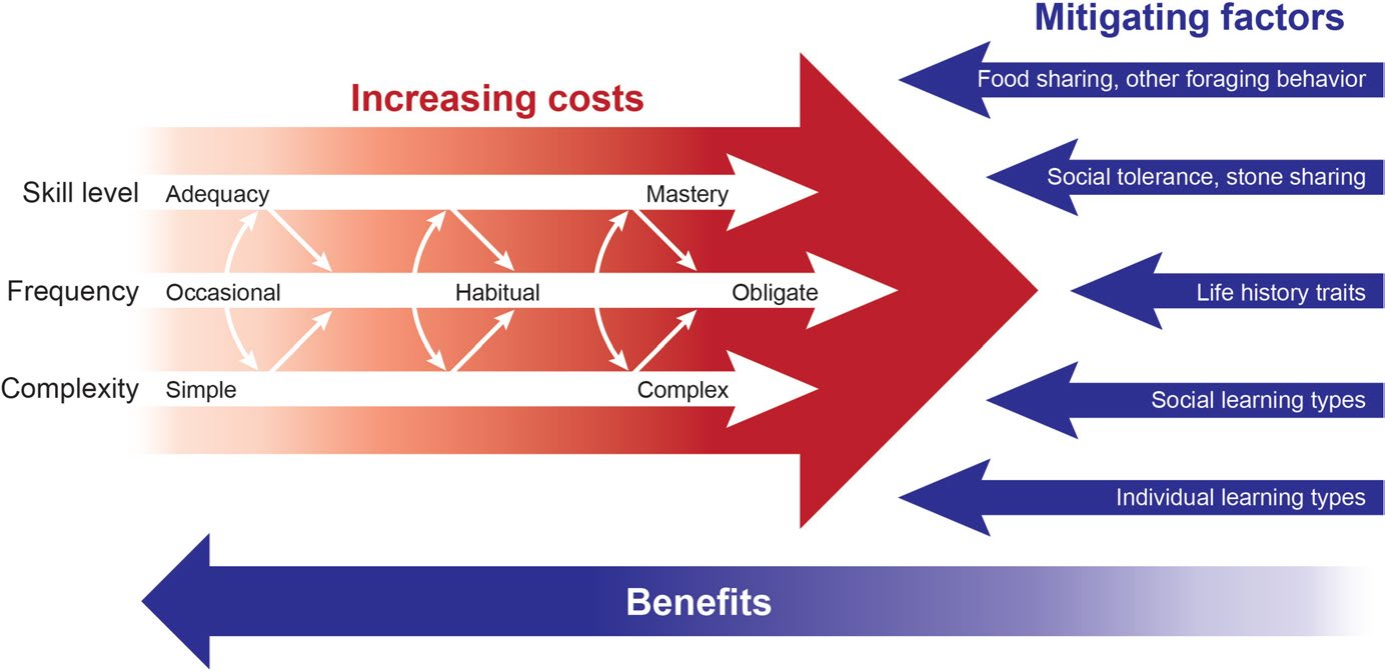
Schematic representation of the general relationship between the factors that increase the relative costs of stone toolmaking, the factors that can potentially mitigate these costs (at the proximate or ultimate level), and the benefits of knapping

If we consider know-how copying origins from the angle of cost-driven estimation, there might be an allure to argue that know-how copying existed for the transmission of early stone tool manufacture. For one, know-how copying is hypothetically useful for transmitting such skills. And at base value, the notion that knapping costs might have created a selection gradient for (improved) know-how copying abilities has some appeal. But, we have argued, these statements have little explanatory power: they do not tell us much of anything about the nature of the relationship between know-how copying and knapping costs, about how know-copying evolved, when it evolved, or about when knapping costs might have created a dependency upon know-how copying. Given the current evidence, it is nearly impossible to draw any conclusion about any of these open questions (Fig. 2). At the present, we know too little about:

- The habituality of toolmaking and/or how dependent different hominins were on knapping for survival (see Karakostis, 2023; Shea, 2017; Toth, 1985),
- The relative magnitude of the costs involved in knapping (see Bamforth & Bleed, 1997),
- The overall cost–benefit profile of knapping behavior,
- The (other) kinds of mitigating factors, e.g., related to life history or social behavior (e.g., Alger et al., 2023; Hrdy, 2009; Opie & Power, 2008; Stout, 2005), that might have instead reduced knapping costs,
- The costs and net benefits of different social learning types/mechanisms (cf. Enquist et al., 2023; Lycett, 2015, 2019; Lycett et al., 2015) and of individual learning (consider, e.g., Eteson et al., 2024; van Schaik & Burkart, 2011),
- And other behavioral domains and pathways that might have been involved or even solely responsible for the evolution of know-how copying variants (e.g., Andersson & Tennie, 2023; Boyd et al., 2011; Fragaszy et al., 2024; Henrich, 2015; Planer, 2017, 2023; Reindl et al., 2016; Richerson & Boyd, 2000; Rolian & Carvalho, 2017; Sterelny, 2022; Timmermann et al., 2024).

Because of such significant ambiguity within our present understanding of these phenomena, we should hesitate to infer too strongly about the role of knapping costs in driving the evolution of know-how copying. The idea that these costs would have necessitated know-how copying in order to be mitigated in the case of early stone tools can be logically discarded. It is also highly doubtful that the first toolmaking hominins already possessed know-how copying abilities, or at least, that they had to employ these abilities for transmitting early stone tool know-how (Tennie et al., 2016, 2017). To the extent that the costs of knapping were a factor in the evolution of know-how copying, we suggest that these abilities either arose de novo only after the Early Acheulean or else that they evolved via some other pathway and were then subsequently enhanced by the costs associated with progressively more complex technological know-how and increasingly habitual and frequent knapping occurrences.

## Data Availability

Data sharing not applicable to this article as no datasets were generated or analyzed during the current study.
